# Association of coal mine dust lung disease with Nodular thyroid disease in coal miners: A retrospective observational study in China

**DOI:** 10.3389/fpubh.2022.1005721

**Published:** 2022-10-28

**Authors:** Feng Zhao, Hongzhen Zhang, Dingfei Ren, Chang-ming Li, Yaqi Gu, Yisong Wang, Dekun Lu, Zongyao Zhang, Qi Lu, Xinxin Shi, Lixin Yang

**Affiliations:** ^1^The First Hospital of Anhui University of Science and Technology (Huainan First People's Hospital), Huainan, China; ^2^Anhui University of Science and Technology College of Medicine, Huainan, China; ^3^Occupational Control Hospital of Huaihe Energy Group, Huainan, China; ^4^Xinhua Hospital, Huainan Xinhua Medical Group, Huainan, China

**Keywords:** coal miners, underground operating environment, coal mine dust lung disease, Nodular thyroid disease, retrospective observational study

## Abstract

**Background:**

Coal dust is a major risk factor for the occupational health of coal miners, and underground workers with coal mine dust lung disease (Coal miners with coal mine dust lung disease (CMDLD) may have a higher risk of developing Nodular thyroid disease (NTD). The aim of this study was to investigate the relationship between coal mine dust lung disease and the development of Nodular thyroid disease in coal miners.

**Methods:**

This was a clinical retrospective observational study that included 955 male coal miners from 31 different coal mining companies in Huainan, Anhui Province, China, who were examined in April 2021 at the Huainan Occupational Disease Prevention and Control Hospital to collect all their clinical physical examination data, including general conditions, laboratory test indices and imaging indices. Based on the presence or absence of Nodular thyroid disease, 429 cases with Nodular thyroid disease were classified as the diseased group and 526 cases without Nodular thyroid disease were classified as the control group. Logistic regression was used to analyse the correlation between the occurrence of Nodular thyroid disease in coal miners, and further single- and multi-factor logistic regression was used to screen the risk exposure factors for Nodular thyroid disease in coal miners.

**Results:**

Age, coal mine dust lung disease (CMDLD), red blood cells (RBC), mean red blood cell volume (MCV), albumin (ALB), albumin/globulin (A/G), indirect bilirubin (IBIL), globulin (GLOB), total bilirubin (TBil) and myeloperoxidase (MPO) were associated with the development of Nodular thyroid disease in coal miners (*p* < 0.05) The results of univariate and multifactorial logistic regression analysis showed that CMDLD (OR:4.5,95%CI:2.79–7.51) had the highest OR and CMDLD was the strongest independent risk exposure factor for the development of Nodular thyroid disease in coal miners.

**Conclusions:**

There is a strong correlation between coal mine dust lung disease and Nodular thyroid disease in underground coal miners, and clinicians need to be highly aware of the high risk of NTD in coal miners with CMDLD and adopt individualized clinical prevention strategies.

## Introduction

Coal is China's main energy resource and about 70% of China's electricity comes from coal-fired power plants, and there are currently >6 million (1) underground miners, which is a large occupational health population. With the world economy growing at a rapid pace, raw coal production and total coal production have increased significantly and the number of underground miners continues to grow (2). The increasing mining and use of coal has led to an increasing number of coal miners being exposed to the health hazards of coal mine dust (3), and the occupational health of coal miners is in urgent need of widespread medical attention.

The lung diseases of coal miners caused by long-term inhalation of coal mine dust are collectively known as coal mine dust lung diseases (CMDLD). CMDLD includes coal workers' pneumoconiosis (CWP), silicosis, bronchiectasis, emphysema and cancer (4, 5). CWP is a classic preventable but not fully curable occupational CMDLD (6), with >4,000 new cases of CWP diagnosed in coal miners each year, and long-term coal dust exposure is the leading cause of death from respiratory disease and complications in coal miners (7–9). Coal dust can cause respiratory diseases in coal miners, but also endocrine diseases, cardiovascular diseases and other multi-system diseases (10–12). Nodular thyroid disease is a common endocrine disorder and a large retrospective study analyzed data (13) from clinical physical examinations of 16,929 medical staff and found that the prevalence of Nodular thyroid disease in men was around 34%.

In this study, we collected clinical examination data from 955 male coal miners operating in underground environments. Four hundred and twenty nine coal miners had Nodular thyroid disease, and their prevalence was as high as 44.9%. Therefore, we hypothesize that the prevalence of Nodular thyroid disease in coal miners is higher than in other occupational groups, which may be related to the long-term exposure of coal miners to coal dust environment, and coal miners with CMDLD may be at a high risk of developing Nodular thyroid disease Coal miners with CMDLD may be at high risk of developing Nodular thyroid disease, but there are no studies on the association between CMDLD and NTD in coal miners. This study investigated the association between coal mine dust lung disease and Nodular thyroid disease in coal miners by retrospectively analyzing clinical examination data from 955 coal miners with underground operations in Huainan, Anhui Province, China, in 2021, with the aim of providing precise prevention strategies for primary prevention of Nodular thyroid disease in coal miners.

## Materials and methods

### Study population

This is a clinical retrospective observational study that collected clinical physical examination data from 955 male coal miners from 31 different coal mining companies in Huainan City, Anhui Province, China, who were examined in April 2021 at the Occupational Disease Prevention and Control Hospital in Huainan City, Anhui Province, China. Inclusion criteria: (1) Male coal miners aged ≥18 years. (2) No serious organic lesions. (3) In addition to the routine physical examination, the physical examination also included thyroid and lung examinations, and the physical examination information was complete and free of defects. Exclusion Criteria: (1) Serious cardiovascular, cerebrovascular, liver, kidney or other serious primary diseases. (2) Those with severe mental disorders or who are unable to cooperate with the medical examination for various reasons. (3) Those with a history of surgery or chemotherapy for malignant tumors. (4) Those with malignant diseases or serious systemic infections. (5) Incomplete thyroid and lung physical examination data. Finally, 955 male coal miners met the study requirements and were included in the observation. All personally identifiable information was encrypted by the researchers, no personal privacy was disclosed, and the ethical requirements of the ethical review committee of the First Affiliated Hospital of Anhui University of Technology (Huainan First People's Hospital) were met.

### Statistical analysis

The study was statistically analyzed using 4.0.3 statistical software. Quantitative data were determined to be normal using the Shapiro normality test, with normally distributed data expressed as ($\bar{x}$ ± s) and independent samples *t*-test for comparison between groups; non-normally distributed data were expressed as M (P_25_, P_75_) and wilcox test for comparison between groups. Qualitative data were statistically described using frequencies (%) and comparisons between groups were made using the χ*2* test or Fisher's exact test. Factors influencing Nodular thyroid disease were analyzed using one-way and multi-way logistic regression analysis. Differences were considered statistically significant when bilateral *p*-values were <0.05.

## Diagnostic criteria

(1) Diagnosis of coal mine dust lung disease: In this study, coal mine dust lung disease refers to a range of lung diseases caused by long-term exposure to coal mine dust, including pneumonia, pneumoconiosis, silicosis, dust-related diffuse fibrosis (DDF) and chronic obstructive pulmonary disease (COPD) (14, 15). Pulmonary imaging is performed on coal miners using Siemens Somatom Definition AS 64-row 128-slice spiral CT, United Imaging UCT580 40-row 40-slice spiral CT, United Imaging UDR770 and UDR260 digital radiographs, and all imaging diagnoses of coal mine dust lung disease are made by two or more qualified imaging physicians.

(2) Diagnosis of Nodular thyroid disease: The diagnosis of Nodular thyroid disease in this study was based on clinical manifestations, laboratory tests and imaging examinations of the coal miners, and the diagnosis of Nodular thyroid disease was in accordance with the diagnostic criteria of the Chinese guidelines for the diagnosis and treatment of Nodular thyroid disease (16). Thyroid ultrasound examination of coal miners using Myriad Resona 7, Myriad DC-8 EXP, Myriad DC-80, Toshiba APLIO 500, Siemens ACUSON Oxana 2,a linear array high frequency probe is used with a probe frequency of 5~15 MHz, where thyroid nodules were classified in accordance with the diagnostic criteria of the 2020 Chinese Guidelines for Ultrasound Risk Stratification of Thyroid Nodules for Malignancy: C-TIRADS (17). According to the C-TIRADS classification criteria, thyroid nodules were classified according to their ultrasound presentation. Thyroid nodules with solid, very hypoechoic, microcalcifications, blurred margins, irregular morphology and vertical growth or extrathyroidal invasion were defined as suspicious malignant features and assigned 1 point each, while comet tail artifacts were defined as benign features and assigned−1 point. Each thyroid nodule was assigned a value and summed, and those with−1 score were classified as category 2 with 0 risk of malignancy; those with 0 score were classified as category 3 with <2% risk of malignancy; those with 1 score were classified as category 4a with 2–9% risk of malignancy; those with 2 scores were classified as category 4b with 10–49% risk of malignancy; those with 3–4 scores were classified as category 4c with 50–90% risk of malignancy; and those with 5 scores and above were classified as category 5 with >90% risk of malignancy 90%. All nodular thyroid diseases were diagnosed by 2 ultrasonographers with the title of attending physician or higher.

## Results

### Baseline clinical characteristics of the participants

**Figure 1:**
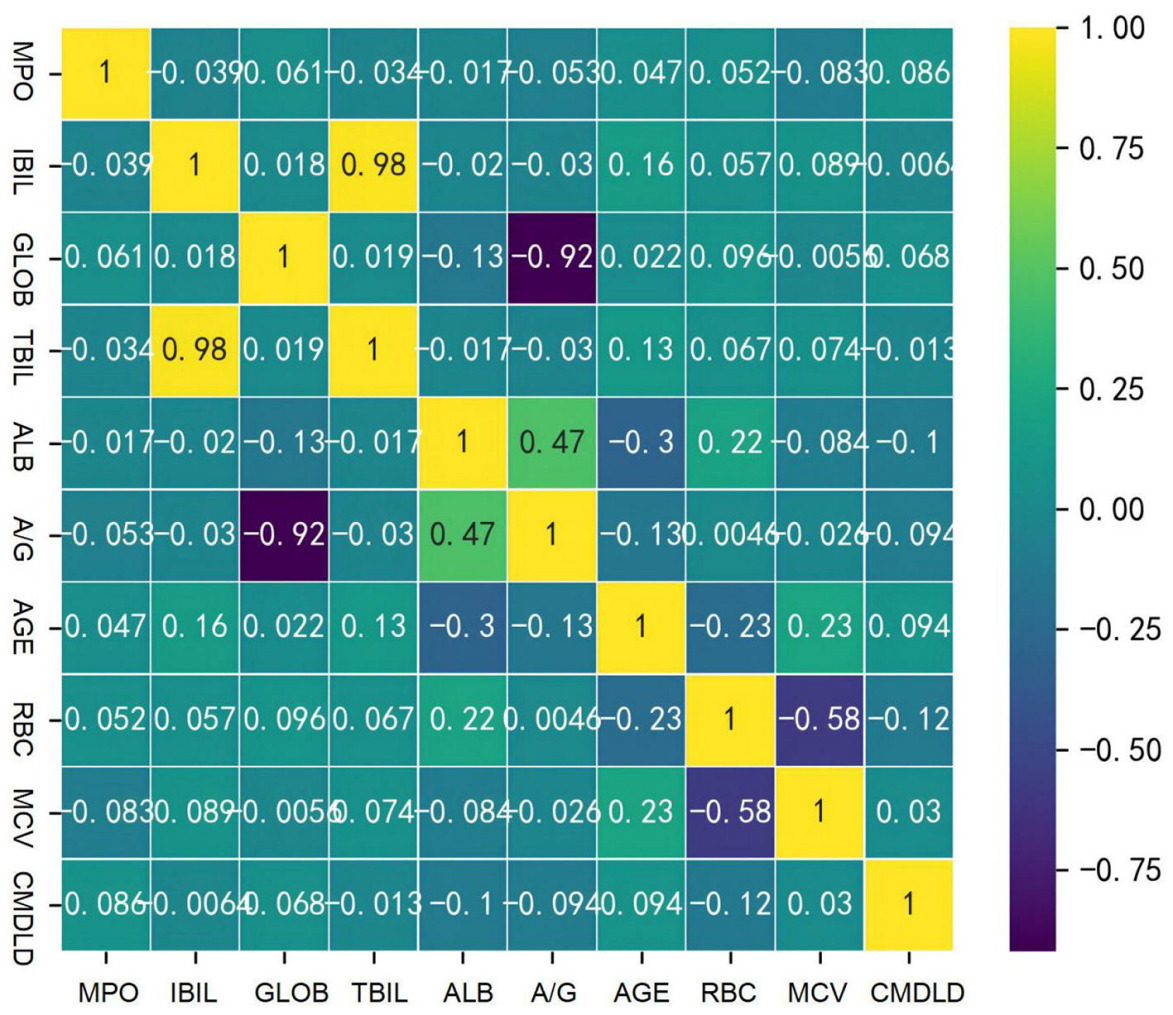
Heat maps of correlation of clinical features in coal miners.

In this study, clinical physical examination data of 955 coal miners were summarized. The number of coal miners with Nodular thyroid disease was 429 and the number of coal miners without Nodular thyroid disease was 526. After statistical test analysis, Age, CMDLD, RBC, MCV, ALB, A/G, IBIL, GLOB, TBil, and MPO were statistically significant between the two groups of coal miners with Nodular thyroid disease, while the remaining variables were not statistically significant between groups (*P*-value > 0.05), as shown in Table 1. We further did correlation heat map for these 10 statistically significant variables. The analysis revealed that CMDLD was an independent predictor and none of the other variables were correlated, while TBIL was definitely correlated with IBIL and A/G was correlated with ALB (Figure 1). As IBIL and A/B were more characteristic, we chose IBIL and A/B instead of TBIL and ALB in order to reduce the interference of confounding factors.

**Table 1:** Baseline clinical characteristics of two groups of coal miners.

**Characteristics**	**Non-Nodular thyroid disease (*n* = 526)**	**Nodular thyroid disease (*n* = 429)**	**Statistic**	***P*** **-value**
CMDLD			52.472	<0.001^***^
0, *n*%	503 (95.63%)	347 (80.89%)		
1, *n*%	23 (4.37%)	82 (19.11%)		
Age, year, M (P_25_, P_75_)	39 (33.25, 48)	43 (36,50)	−4.479	<0.001^***^
LDL-C, mmol/L, M (P_25_, P_75_)	2.76 (2.36, 3.2)	2.84 (2.39, 3.36)	−1.792	0.073
HDL-C, mmol/L, M (P_25_, P_75_)	1.17 (1.03, 1.38)	1.16 (1.02, 1.35)	0.460	0.646
WBC, 10^9^/L, M (P_25_, P_75_)	6.51 (5.66, 7.49)	6.5 (5.55, 7.74)	−0.500	0.617
RBC, 10^12^/L, M (P_25_, P_75_)	4.98 (4.76, 5.22)	4.91 (4.7, 5.15)	2.680	0.007^**^
MCV, fL, M (P_25_, P_75_)	92.2 (89.93, 94.6)	92.6 (90.5, 95.1)	−2.116	0.034^*^
ALB, g/L, M (P_25_, P_75_)	47.39 (45.88, 48.66)	46.62 (44.9, 48.08)	5.080	<0.001^***^
A/G, M (P_25_, P_75_)	1.67 (1.53, 1.8)	1.6 (1.44, 1.73)	4.820	<0.001^***^
GGT, U/L, M (P_25_, P_75_)	28.7 (20.17, 43.85)	30.95 (21.6, 48.43)	−1.830	0.067
ALT, U/L, M (P_25_, P_75_)	21.1 (14.78, 30.8)	22.05 (15.57, 31)	−0.957	0.339
AST/ALT, M (P_25_, P_75_)	1.01 (0.79, 1.3)	1 (0.79, 1.31)	0.622	0.534
IBIL, umol/L, M (P_25_, P_75_)	9.5 (7.4, 12.3)	9.85 (8.22, 12.88)	−2.181	0.029^*^
ALP, U/L, M (P_25_, P_75_)	72.95 (60.9, 86.93)	74.55 (64.5, 84.8)	−1.309	0.191
GLOB, g/L, M (P_25_, P_75_)	28.42 (26.5, 30.47)	29.32 (27.07, 31.62)	−3.652	<0.001^***^
DBil, umol/L, M (P_25_, P_75_)	3.8 (2.88, 5)	3.9 (3.1, 5.1)	−1.480	0.139
TBil, umol/L, M (P_25_, P_75_)	13.3 (10.5, 17.1)	13.85 (11.4, 17.7)	−2.107	0.035^*^
TP, g/L, M (P_25_, P_75_)	75.5 (73.3, 78.2)	75.8 (73.11, 78.49)	−0.268	0.789
FBG, mmol/L, M (P_25_, P_75_)	6 (5.67, 6.47)	6 (5.67, 6.5)	−0.461	0.645
BUA, μmol/L, M (P_25_, P_75_)	330.05 (277.15, 380.7)	328.8 (282, 379.15)	−0.404	0.686
CYFRA21-1, ng/ml, M (P_25_, P_75_)	2.21 (1.96, 2.52)	2.22 (2, 2.51)	−1.155	0.248
MPO, U/ml, M (P_25_, P_75_)	68.18 (53.83, 89.52)	74.63 (60.8, 98.25)	−3.829	<0.001^***^
Lp-PLA2, M (P_25_, P_75_)	336.15 (252.28, 432.65)	323.18 (241.2, 405.54)	1.649	0.099

HDL-C, High-density lipoprotein cholesterol; LDL-C, Low-density lipoprotein cholesterol; ALB, Serum albumin; GLOB, globulin; IBIL, Indirect bilirubin; Lp-PLA2, Lipoprotein-associated phospholipase; A2,MPO, Myeloperoxidase; CYFRA21-1, Cytokeratin 19 fragment; MCV, Mean red blood cell volume; RBC, Red blood cells; WBC, Leukocytes; TBil, Total bilirubin; A/G, Albumin/globulin; GGT, Glutamyl transpeptidase; ALT, Glutathione aminotransferase; AST/ALT, Glutathione/glutathione transaminase; ALP, Alkaline phosphatase; DBil, Direct bilirubin; TP, Total protein; FBG, Plasma fibrinogen; BUA, Blood uric acid.

* P < 0.05,

** P < 0.01,

*** P < 0.001.

### Univariate and multifactorial logistic regression analysis of nodular thyroid disease in coal miners

**Figure 2:**
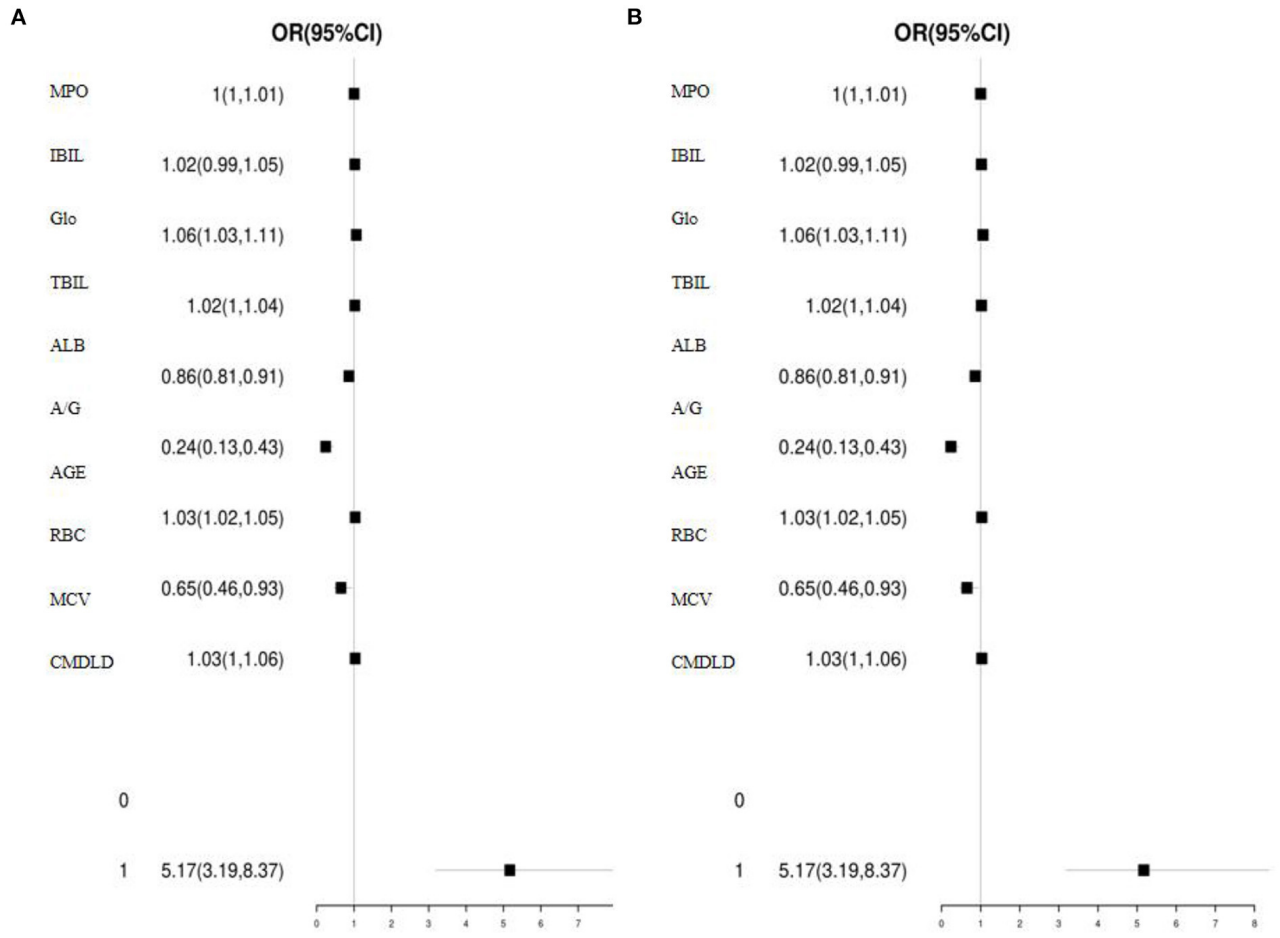
**(A,B)** Univariate and multifactorial analysis of forest plots for the occurrence of nodular thyroid disease in coal miners.

Univariate and multivariate logistic regression analyses were performed using whether the patient had Nodular thyroid disease as the dependent variable (normal-0, abnormal-1) and whether they had coal mine dust lung disease as the independent variable (normal-0, abnormal-1) (Table 2). The results of the univariate analysis showed that the differences in having CMDLD, MPO, GLOB, ALB, A/G, AGE and RBC were all statistically significant (*p* < 0.05). All parameters included in the above univariate analysis were further analyzed by multi-factor logistic regression, with OR values suggesting the relative risk of developing Nodular thyroid disease in coal miners. The results showed that CMDLD (OR: 5.11, 95% CI: 3.15–8.29), MPO (OR: 1.00, 95% CI: 1.00–1.01), GLOB (OR: 1.06, 95% CI: 1.02–1.10), ALB (OR: 0.85, 95% CI: 0.80–0.90), A/G (OR. CMDLD, GLOB, and AGE were all independent risk factors for the development of Nodular thyroid disease in coal miners. In addition, the OR for CMDLD was the highest of all independent risk factors (Figure 2). Therefore, having CMDLD is the strongest independent risk exposure factor for Nodular thyroid disease in coal miners (*p* < 0.0001) and clinicians should focus on the risk of Nodular thyroid disease in coal miners with CMDLD.

**Table 2:** Univariate and multifactorial logistic regression analysis of the occurrence of thyroid disease in coal miners.

**Variables**	***N***	**Univariate analysis**		**Multivariate analysis**	
		**OR (95%CI)**	***P*** **-value**	**OR (95%CI)**	***P*** **-value**
CMDLD	955	/	/	/	/
0	850	/	/	/	/
1	105	5.17 (3.19~8.37)	*P* < 0.0001^***^	5.11 (3.15~8.29)	*P* < 0.0001^***^
MPO	955.0	1.00 (1.00~1.01)	0.01^*^	1.00 (1.00~1.01)	0.01^*^
IBIL	955.0	1.02 (0.99~1.05)	0.11	1.02 (0.99~1.05)	0.12
GLOB	955.0	1.06 (1.03~1.11)	*P* < 0.0001^***^	1.06 (1.02~1.10)	*P* < 0.0001^***^
TBIL	955.0	1.02 (1.00~1.04)	0.13	1.02 (1.00~1.04)	0.11
ALB	955.0	0.86 (0.81~0.91)	*P* < 0.0001^***^	0.85 (0.80~0.90)	*P* < 0.0001^***^
A/G	955.0	0.24 (0.13~0.43)	*P* < 0.0001^***^	0.26 (0.14~0.47)	*P* < 0.0001^***^
Age	955.0	1.03 (1.02~1.05)	*P* < 0.0001^***^	1.03 (1.02~1.05)	*P* < 0.0001^***^
RBC	955.0	0.65 (0.46~0.93)	0.02^*^	0.61 (0.42~0.87)	0.01^*^
MCV	955.0	1.03 (1.00~1.06)	0.05	1.03 (1.00~1.06)	0.06

* P < 0.05,

*** P < 0.001.

All indicate significant differences compared between groups.

## Discussion

Coal dust is the most significant risk factor for occupational health risks to coal miners (18). McBean et al. (14) suggested that the majority of coal mine dust lung diseases are associated with coal dust exposure. Not only that, but several researchers (7, 19) have found that coal dust is not only a risk factor for coal miners to develop coal mine dust lung disease, but also a high risk factor for coal miners to develop Nodular thyroid disease. Coal miners exposed to chronic coal dust are at high risk of Nodular thyroid disease, however, the association between coal mine dust lung disease (CMDLD) and Nodular thyroid disease in coal miners is currently unclear, and investigating the risk factors for Nodular thyroid disease in coal miners is of great significance for the primary prevention of Nodular thyroid disease in coal miners. In this study, a retrospective observational study based on clinical examination data of 955 male coal miners from 31 different coal mining companies in Huainan, Anhui Province, China, found that CMDLD was the strongest risk factor for the development of Nodular thyroid disease in coal miners and that clinicians should pay more attention to the development of Nodular thyroid disease in coal miners with CMDLD and take early and effective preventive strategies.

Nodular thyroid disease is one of the most common non-communicable diseases in developing countries (20). Because thyroid disorders rarely lead to serious life-threatening conditions, they are often overlooked (21), leading to their progression to malignant levels. In recent years, Nodular thyroid disease has been on the rise, with the overall prevalence of Nodular thyroid disease among adults over 18 years of age in China reaching 50%, according to the latest survey data from 2020 (22, 23). Statistical analysis of the data in this study found that 955 male coal miners had a 44.9% chance of developing Nodular thyroid disease, and that the prevalence of Nodular thyroid disease among male coal miners working underground was significantly close to the overall prevalence. In a previous study, Koeger et al. (24) found that middle-aged male coal miners with occupational exposure to coal dust silica developed not only silicosis but also Graves' disease, an autoimmune disorder. This study was limited by the small sample size and therefore only identified Graves' disease as a cause of Nodular thyroid disease due to coal dust exposure and did not investigate the association between Graves' disease and silicosis. This study collected clinical examination data from 955 male coal miners from 31 different coal mining companies in Huainan, Anhui Province, China. The large sample size and the wide range of data sources in this study have reduced the bias of the study due to insufficient sample size. In addition, the 70 clinical physical examination variables collected in this study (including general information, laboratory test indicators, and imaging test indicators) were analyzed by univariate and multifactorial logistic regression, and after multifactorial analysis, CMDLD (OR = 5.21, *p* < 0.0001), GLOB (OR = 1.06, *p* < 0.0001), Age (OR = 1.03, *p* < 0.0001) were found to be 0.0001 as independent risk factors for the development of Nodular thyroid disease in coal miners, with CMDLD having the highest OR, much greater than the other two variables, indicating that CMDLD is the highest risk exposure factor for the development of Nodular thyroid disease in coal miners, and is statistically significant. Therefore, focusing on screening for Nodular thyroid disease in coal miners with CMDLD and adopting targeted prevention strategies are important measures to effectively reduce the occurrence of Nodular thyroid disease in coal miners.

There is a lack of research on the mechanism of the relationship between CMDLD and the development of Nodular thyroid disease in coal miners. This study suggests that there may be two main associations. Firstly, when exposed to coal dust for long periods of time, the body adjusts cortisol, thyroid hormone and insulin levels for protective adaptive regulation, and when endocrine gland function reserves are depleted and the body's ability to adapt decreases, coal miners are prone to develop pneumoconiosis (25) and consequently Nodular thyroid disease with abnormal thyroid hormones. Tsukatani et al. (26) found that silicosis caused calcification of paratracheal and upper mediastinal lymph nodes in patients with papillary thyroid carcinoma (PTC), and later surgical pathology demonstrated the presence of both silica nodules and microtransformations of PTC in the paratracheal lymph nodes of patients with PTC; therefore, the silica component of coal dust may be a causative factor between CMDLD and Nodular thyroid disease. Second, an immunological and neurological analysis of the pathogenesis of CMDLD may better explain the existence of a relationship between CMDLD and Nodular thyroid disease in coal miners. The direct toxic effects of silica and monosilicic acid in coal dust on the lungs may be accompanied by an attack on other organs of the organism (27). At the same time, secondary hypoxia due to dust-silica lung may result in inadequate stimulation and excretion of certain hormones, leading to dysfunction of the hypothalamic-pituitary-thyroid axis, which in turn leads to the development of thyroid disorders.

However, there are some limitations that need to be taken into account when interpreting the results of this study. First, the data we collected on physical examinations of coal miners were missing data on coal miners' history of alcohol consumption, smoking, family history of Nodular thyroid disease, body mass index, years of underground work, number of shifts per month and some thyroid hormone blood biochemical indicators, variables that may be associated with the occurrence of Nodular thyroid disease in coal miners. Secondly, due to the fixed nature of the medical examination package, we were unable to obtain further pathological follow-up of the association between Nodular thyroid disease and coal mine dust lung disease, which prevented an in-depth investigation into the pathogenesis of CMDLD and NTD. In the future, we will conduct experimental studies to further investigate the mechanisms by which coal dust causes Nodular thyroid disease and CMDLD in animals. Thirdly, the population in this study was exclusively from Huainan, Anhui Province, China, and no data from other coal miners of different geographical or ethnic origin were included, which may have biased the results of the study. In the next step, we will expand the sample size collection to include data from coal miners from different regions, countries and ethnicities in China, so that this study can have a wider clinical utility.

This study found a high correlation between coal mine dust lung disease and the development of Nodular thyroid disease in coal mine workers, and that coal mine dust lung disease is a high risk factor for the development of Nodular thyroid disease in coal mine workers. With the exception of thyroiditis, any benign change in Nodular thyroid disease is associated with an increased risk of thyroid cancer (28). Nodular thyroid disease has a serious impact on the occupational health of coal miners. The disease can place additional physical, mental and financial burdens on coal miners and their families, and may even shorten life expectancy, and requires the joint attention of government departments and the medical community.

## Conclusion

Mine miners who are chronically exposed to working in a coal dust environment are at high risk of developing nodular thyroid disease. It is worth noting that Age, CMDLD, RBC, MCV, ALB, A/G, IBIL, GLOB, TBil, and MPO are influential factors for the development of nodular thyroid nodular disease in coal miners, with CMDLD being the strongest risk exposure factor for nodular thyroid disease in coal miners. Therefore, clinicians should be highly concerned about the high risk of nodular thyroid in coal miners with CMDLD and take early and individualized preventive and therapeutic measures, which are of great clinical value to improve the occupational health and safety of coal miners with thyroid.

## Data availability statement

The raw data supporting the conclusions of this article will be made available by the authors, without undue reservation.

## Ethics statement

Ethical review and approval was not required for the study on human participants in accordance with the local legislation and institutional requirements. Written informed consent from the patients was not required to participate in this study in accordance with the national legislation and the institutional requirements.

## Author contributions

FZ and HZ contributed to the writing of the manuscript, design of the study, and statistical analysis. DR and C-mL contributed to the conception and design of this study. YG, YW, DL, and ZZ contributed to data retrieval and manuscript review. QL, XS, and LY contributed to data collection and data collation. All authors made significant contributions to the research process of this manuscript, read, and approved the submitted manuscript.

## Funding

The authors carried out all the work in this study with the support of the Department of the Huainan Municipal Committee of Anhui Province and the Huainan Science and Technology Bureau. This study was funded by the Key Research Project of Anhui Provincial Education Department (No. KJ2019A0094), the Key Research Project of Anhui Provincial Education Department (No. KJ2019A0095), and the Research Project Plan of Bengbu Medical College (No. BYKY2019318ZD). This work was supported by the Huainan City Occupational Disease Control Institute, China, the School of Medicine of Anhui University of Technology, and the First People's Hospital affiliated to Anhui University of Technology (Huainan First People's Hospital).

## Conflict of interest

The authors declare that the research was conducted in the absence of any commercial or financial relationships that could be construed as a potential conflict of interest.

## Publisher's note

All claims expressed in this article are solely those of the authors and do not necessarily represent those of their affiliated organizations, or those of the publisher, the editors and the reviewers. Any product that may be evaluated in this article, or claim that may be made by its manufacturer, is not guaranteed or endorsed by the publisher.

## References

[1] Liu FD Pan ZQ Liu SL Chen L Ma JZ Yang ML. The estimation of the number of underground coal miners and the annual dose to coal miners in China. Health Phys. (2007) 93:127–32. 10.1097/01.HP.0000261600.29366.b4 17622817

[2] Liu FD Pan ZQ Liu SL Chen L Chen L Wang CH. The estimation of the number of underground coal miners and normalization collective dose at present in China. Radiat Prot Dosimetry. (2017) 174:302–7. 10.1093/rpd/ncw146 27342453

[3] Bridbord K Costello J Gamble J Groce D Hutchison M Jones W. Occupational safety and health implications of increased coal utilization. Environ Health Perspect. (1979) 33:285–302. 10.1289/ehp.7933285 540621 PMC1638124

[4] Sun Y Kinsela AS Cen X Sun S Collins RN Cliff DI. Impact of reactive iron in coal mine dust on oxidant generation and epithelial lung cell viability. Sci Total Environ. (2022) 810:152277. 10.1016/j.scitotenv.2021.152277 34902414

[5] Laney AS Weissman DN. Respiratory diseases caused by coal mine dust. J Occup Environ Med. (2014) 56:S18–22. 10.1097/JOM.0000000000000260 25285970 PMC4556416

[6] Perret JL Miles S Brims F Newbigin K Davidson M Jersmann H. Respiratory surveillance for coal mine dust and artificial stone exposed workers in Australia and New Zealand: a position statement from the thoracic society of Australia and New Zealand. Respirology. (2020) 25:1193–202. 10.1111/resp.13952 33051927 PMC7702073

[7] Liu H Tang Z Yang Y Weng D Sun G Duan Z. Identification and classification of high risk groups for Coal Workers' Pneumoconiosis using an artificial neural network based on occupational histories: a retrospective cohort study. BMC Public Health. (2009) 9:366. 10.1186/1471-2458-9-366 19785771 PMC2760532

[8] Wang D Yang M Liu Y Ma J Shi T Chen W. Association of silica dust exposure and cigarette smoking with mortality among mine and pottery workers in China. JAMA Netw Open. (2020) 3:e202787. 10.1001/jamanetworkopen.2020.2787 32286660 PMC7156992

[9] Rahman MM Bibi S Rahaman MS Rahman F Islam F Khan MS. Natural therapeutics and nutraceuticals for lung diseases: traditional significance, phytochemistry, and pharmacology. Biomed Pharmacother. (2022) 150:113041. 10.1016/j.biopha.2022.113041 35658211

[10] Heymer B. [On combined incidence of lung silicosis, fibro-pancarditis and fibrous atrophy of the thyroid gland]. Int Arch Arbeitsmed. (1966) 22:20–6. 10.1007/BF00390448 5957907

[11] Parks CG Conrad K Cooper GS. Occupational exposure to crystalline silica and autoimmune disease. Environ Health Perspect. (1999) 107:793–802. 10.1289/ehp.99107s5793 10970168 PMC1566238

[12] Wu Q Han L Xu M Zhang H Ding B Zhu B. Effects of occupational exposure to dust on chest radiograph, pulmonary function, blood pressure and electrocardiogram among coal miners in an eastern province, China. BMC Public Health. (2019) 19:1229. 10.1186/s12889-019-7568-5 31488099 PMC6728990

[13] Chen P Wang M. A survey on the incidence of thyroid disease among 16929 medical workers in the western region. J Baotou Med Coll. (2016) 32:9–11.

[14] McBean R Tatkovic A Edwards R Newbigin K. What does coal mine dust lung disease look like? a radiological review following re-identification in Queensland. J Med Imaging Radiat Oncol. (2020) 64:229–35. 10.1111/1754-9485.13007 32048474

[15] McBean R Newbigin K Dickinson S Edwards R. Radiological appearance of coal mine dust lung diseases in Australian workers. J Med Imaging Radiat Oncol. (2018) 62:794–7. 10.1111/1754-9485.12821 30341807

[16] Chinese Medical Association E, China Thyroid Diseases Diagnosis and Treatment Guidelines Writing Group. Chinese guidelines for the diagnosis and treatment of thyroid diseases - laboratory and adjunctive tests for thyroid diseases. Chin J Intern Med. (2007) 46:697–702. 10.3760/j.issn:0578-1426.2007.08.038

[17] Zhou J Yin L Wei X Zhang S Song Y Luo B. 2020 Chinese guidelines for ultrasound malignancy risk stratification of thyroid nodules: the C-TIRADS. Endocrine. (2020) 70:256–79. 10.1007/s12020-020-02441-y 32827126

[18] Liu T Liu S. The impacts of coal dust on miners' health: a review. Environ Res. (2020) 190:109849. 10.1016/j.envres.2020.109849 32763275

[19] Fualal J Ehrenkranz J. Access, availability, and infrastructure deficiency: the current management of thyroid disease in the developing world. Rev Endocr Metab Disord. (2016) 17:583–9. 10.1007/s11154-016-9376-x 27565137

[20] Singal AG Mukherjee A Elmunzer BJ Higgins PD Lok AS Zhu J. Machine learning algorithms outperform conventional regression models in predicting development of hepatocellular carcinoma. Am J Gastroenterol. (2013) 108:1723–30. 10.1038/ajg.2013.332 24169273 PMC4610387

[21] Wu Y Rao K Liu J Han C Gong L Chong Y. Machine learning algorithms for the prediction of central lymph node metastasis in patients with papillary thyroid cancer. Front Endocrinol. (2020) 11:577537. 10.3389/fendo.2020.577537 33193092 PMC7609926

[22] Zongyan S Weiping T. Current status, countermeasures and challenges in the prevention and treatment of thyroid diseases in China. Diagn Theory Pract. (2020) 19:329–33.

[23] Zongyan S. Efficacy and safety of long-term universal salt iodization: epidemiological evidence from 31 provinces in mainland China. Int J Endocrinol Metab. (2020) 40:314. 10.3760/cma.j.issn.1673-4157.2020.05.102 32075540

[24] Koeger AC Alcaix D Rozenberg S Bourgeois P. Graves' disease after silica dust exposure. J Rheumatol. (1996) 23:202. 8838545

[25] Kulkybaev GA AKh A Baimanova AM. [The body's hormonal balance in miners at the coal mines]. Med Tr Prom Ekol. (1998) 24:21–4. 9855742

[26] Tsukatani T Niwa H Komori T Yoneyama T Tsuji H Michigishi T. Superior mediastinal lymphadenopathy by silicosis mimicking metastasis of papillary thyroid carcinoma - case report and literature review. Auris Nasus Larynx. (2020) 47:1054–7. 10.1016/j.anl.2019.12.003 31899060

[27] Ruse M Dascălu R Suciu I Zergreanu O. [Participation of gonads in pulmonary silicosis]. Z Gesamte Inn Med. (1976) 31:281–4. 183394

[28] Schiffmann L Kostev K Kalder M. Association between various thyroid gland diseases, TSH values and thyroid cancer: a case-control study. J Cancer Res Clin Oncol. (2020) 146:2989–94. 10.1007/s00432-020-03283-x 32518973 PMC11804546

